# 

**Published:** 1889-03

**Authors:** 


"Receipts" will appear in our next.

